# Synthesis of nanogeopolymer adsorbent and its application and reusability in the removal of methylene blue from wastewater using response surface methodology (RSM)

**DOI:** 10.1038/s41598-024-70284-y

**Published:** 2024-09-04

**Authors:** E. M. Abdel Hamid, H. M. Aly, K. A. M. El Naggar

**Affiliations:** 1 Chemical Engineering Department, Egyptian Academy for Engineering and Advanced Technology (EAEAT), Km 3 Cairo-Belbeis, Desert Road, PO box 3056, Cairo, Egypt; 2 Chemical Engineering Department, National Research Centre, Cairo, Egypt

**Keywords:** Box-Behnken design, RSM, Adsorption, Nanogeopolymer, Methylene blue, Optimization, Chemical engineering, Surface chemistry, Sustainability, Pollution remediation

## Abstract

Organic dyestuff are mostly toxic compounds that pose serious dangers to the environment. Adsorption using low-cost adsorbents is the most favorable method for its economic aspects. Recently, geopolymers have been introduced as an effective adsorbent for dyes and heavy metals. In this investigation, the synthesis of geopolymers from fired brick waste (Homra) was studied with full characterization using X-ray Diffraction, Fourier Transform Infrared Spectroscopy, Brunauer–Emmett–Teller, Energy dispersive X-ray, Scanning electron microscope tests and Transmission electron microscopy. The synthesized nano-Homra geopolymer (NHGP) was then subjected to the removal of one of the most used basic dyes, Methylene Blue (MB). Adsorption optimization was applied using Response surface methodology to study dye adsorption by the synthesized nano-geopolymer. The independent variables studied were: temperature, contact time, and concentration of dye in the elimination process, which were varied in the range of (25–60 ℃), (10–180 min), and (20–300 mg/L) respectively. The results obtained from ANOVA indicated that the maximum removal efficiency of 95% and adsorption capacity of 80.65 mg/g at a temperature of 59 ℃, contact time of 163 min, and an initial concentration of 254 mg/L. The results showed that the data obtained from the adsorption of MB onto NHGP was compatible with the Pseudo second order (R^2^ = 0.9838) and Langmuir isotherm model (R^2^ = 0.9882).

## Introduction

One of the most important issues facing humanity today is water pollution, which is getting worse every day as a result of massive industrial expansion taking place all over the world^1^. The main sources of toxins for the environment in general and water in particular are mostly the organic dyestuffs used in various industries, such as plastics, cosmetics, painting, varnish, and textiles^2^. Many of the operations in these manufacturing sectors include the use of water, and a sizable portion of the tainted water is released into the environment as wastewater^3^. Because organic dyes are highly persistent, poisonous, and nonbiodegradable, they pose a threat to living organisms in water and have the power to alter the water's properties even at low concentrations^4^. Artificial dyes can alter the color of water and block off sunlight, which interferes with aquatic plants' ability to photosynthesize. Furthermore, artificial coloring raises the water's BOD, COD, and particles in suspension^5–8^. Nowadays, more than 700,000 tons of synthetic dyes (about 10,000 commercial varieties) are produced each year^9^. Not only are most dyes toxic and carcinogenic but decontaminating dye-containing effluents can also be a useful means of reusing clean water resources from the treatment of industrial wastewater, which assists in addressing one of humanity's most pressing concerns: water scarcity. Consequently, the advancement of wastewater treatment technology holds significant importance since it has the potential to augment the world's water supply. The flocculation precipitation method, electrolysis, membrane filtration, oxidation, biological degradation, and adsorption, among others, are the techniques that are frequently used in the treatment of industrial wastewater ^10–15^. Because of adsorption's many benefits, including its high efficiency, affordability, and simplicity, it has gained continual attention in the domains of dye removal. Many materials, including activated carbon, coal ash, resins, zeolite, metal oxides, kaolin, magnetic material adsorbents, polypyrrole-functionalized adsorbents, and molecular sieves, have been used as adsorbents in the present day with positive results ^16–23^.

Inorganic polymers known as geopolymers are prepared through the dissolution, polycondensation, and structural reorganization of low-cost aluminosilicate minerals, such as metakaolin, fly ash, and slag. At the moment, geopolymer materials have been thoroughly studied and used in a number of industrial applications because of their favorable chemical and physical characteristics, including excellent mechanical qualities, high durability, great chemical stability, potent immobilization, and strong heat resistance^24^. Due to the fact that industrial wastes like fly ash, silica fume, slag, glass slag, and biomass ash are abundant, readily available, and less likely to deplete the benchmark raw material, metakaolin, as well as their notable environmental benefits, the synthesis of geopolymers from these materials is thought to be a cost-effective option^25–27^. In terms of structure and functionality, geopolymer can be thought of as a three-dimensional aluminosilicate structure with an amorphous or semi-crystalline microstructure, similar to zeolite. It is possible for geopolymerization to occur at temperatures below 100 ◦C, which simplifies and uses less energy and water than zeolites in the production of geopolymer. Low porosity, on the other hand, hinders the industrial applicability of geopolymers. Consequently, to enhance the ability of geopolymers to remove colors from wastewater, it is imperative that their chemical and physical properties be updated on a regular basis. According to Barbosa et al., soybean oil was added to the reaction system of 1SiO_2_:0.25Al_2_O_3_:5.2H_2_O:0.5 K_2_O to direct the mesostructured. To create mesoporous geopolymer, which has a higher adsorption capacity than those made without oil because of its optimized pore characteristics, a friable solid monolith was first created hydrothermally. It was then crushed and cleaned with hexane to remove the oil from the material^28^.

In recent years, there has been increasing emphasis on the removal of dyes from these materials^2,29–31^. Geopolymer's chemical structure is an aluminosilicate framework that is negatively charged and well-adjusted by some cations e.g. (Na^+^, K^+^, or Cs^+^), which can be swapped out for cations in a solution. As a result, this characteristic serves as the foundation for the adsorption of cationic dyes^32,33^. Even though the use of geopolymer as an adsorbent in treating wastewater is still in its beginnings, some important variables have been thoroughly studied, including temperature, pH, the amount of absorbent, and the initial concentration of pollutants. Numerous initiatives have been put in place to boost the capability of adsorption. Ash-based geopolymer materials spheres were manufactured by Novais et al. using a suspension-solidification technique. Because of its superior diffusion behavior and high surface area, methylene blue has an adsorption capacity of 79.7 mg/g^34^. Jin et al. also fabricated a metakaolin-based geopolymer (MG) that served as a quick removal for nickel ions and methylene blue (MB) to aid in the separation and recycling of adsorbents^35^. Li et al. created reusable, porous geopolymer adsorbents with a high adsorption efficiency of 97.8% for the treatment of dye wastewater^36^. According to Hua et al., procion red can be effectively removed from synthetic wastewater by using magnetic geopolymer as an adsorbent for water decolorization^37^. Ali et al. studied employing Fe_3_O_4_ magnetic nanoparticle adsorbents in a batch method to remove the anionic azo dye from wastewater. The impact of trembling speed, time, and adsorbent dose on the removal effectiveness of the dye has been investigated^38^. The sepals of waste tomato plants were used to develop a new green nanocatalyst (GNC) using a thermal approach. The effectiveness of this GNC in reducing the chemical oxygen demand (COD) during a photocatalytic procedure with simulated petroleum refinery liquid waste was studied by Khader et. al.^39^. Also, Khader et al. assessed the efficacy of the adsorption treatment approach in eliminating phenol and acetone in wastewater from industries^40^.

In this work, a novel nano geopolymer was developed for the treatment of methylene blue MB in wastewater, based on the high value-added usage of burnt clay brick waste (Homra). XRD, FTIR, SEM, and BET were used to examine the microstructural characteristics. The RSM method was used to optimize the most effective adsorption parameters at the optimum pH value, and repeated experiments were used to confirm the correctness of the optimized prediction model. According to the findings, nano Homra -geopolymer (NHGP) is a powerful adsorbent that has great potential for eliminating MB from wastewater. The effectiveness of geopolymers' adsorption in eliminating organic contaminants is contingent upon ionic interactions. The positively charged part of methylene blue, one of the most well-known cationic dyes, the cationic part of the dye may trade sites with the geopolymer structure's Na^+^ charge-balancing cations^41^. In addition to standard isotherm and kinetic modeling, the suggested adsorbent underwent concurrent comprehensive characterization, regeneration, reusability, and actual wastewater treatment studies. This is in line with the ideas of sustainable and green development, which emphasize using solid waste to create high-value products and using waste to treat poisoning.

## Materials and methodology

### Raw materials

Fired clay brick waste (Homra) was used as a good substitute for the calcined kaolin which is less expensive. The waste was sieved using mesh size 200, the undersize was used in the preparation of the nano geopolymer. Sodium hydroxide and sodium silicate were used as an alkaline activator in the preparation of geopolymer. They were purchased from El Shark El-Awsat company for chemicals, in Cairo, Egypt. The chemical composition and the mineralogical analysis of Homra were determined using the X-ray fluorescence technique (Axios, panalytical 2005, wavelength dispersive sequential spectrometer machine) and X-ray diffraction Brukur D8 advanced computerized apparatus respectively.

### Methylene blue

Methylene Blue, Methylthioninium chloride, (MB), is a formal derivative of phenothiazine. It is a dark green powder that yields a blue solution in water. The hydrated form has 3 molecules of water per unit of methylene blue.

**Figure 1:**
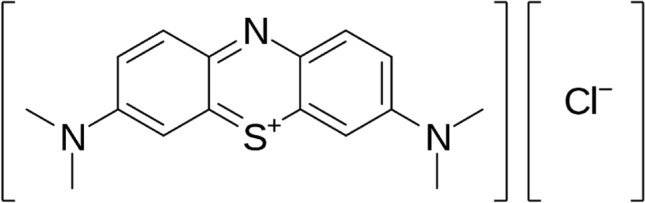
Methylene Blue Structure.

The MB was obtained from RFCL LIMITED with 99% purity, its chemical formula is C_16_H_18_ClN_3_S and its structure is presented in Fig. 1.

### Alkaline activator preparation

The pellets of sodium hydroxide (99%) were dissolved in water to prepare 12 M of an aqueous solution of NaOH. The prepared solution was cooled at room temperature and then mixed with sodium silicate solution (16.7% Na_2_O, 30.3% SiO_2,_ and 53% H_2_O) to produce the alkaline activator. The ratio of Na_2_SiO_3_/ NaOH is 2.5, this mixture was prepared before the synthesis of the geopolymer adsorbent about 24 h^9^.

### Preparation of the geopolymer adsorbent

The aluminum silicates that were present in the Homra waste were mixed with the alkaline activator solution with a solid–liquid (S/L) mass ratio (3/1) using a mechanical mixer to obtain a homogenous mixture. The geopolymer slurry was placed in a cubic iron mold with dimensions (5cm x 5cm). The cubic mold was vibrated using an electric mechanical vibrator to remove any air voids. The sample was kept in the mould for 24 h then de-molded to be cured at room temperature for 28 days. The synthetic geopolymer was crushed and ground to particle size less than100 nm.

### Characterization of the adsorbent

The mineralogical analysis was performed on the synthetic geopolymer with X-ray diffraction to determine the crystalline phases (XRD) using BRUKER apparatus for XRD, Axs, D8- ADVANCE (Germany 2001). Brunauer- Emmett- Teller (BET) analysis was used to determine the surface area presence in the synthetic geopolymer and pore size distribution^42^. BET was performed in the presence of nitrogen adsorption–desorption isotherm analysis at a temperature of 77 K using a 3 Flex 3500 microscopic model analyzer. Scanning electron microscope (SEM) analysis was accomplished to determine the prepared adsorbent morphology^43^ using JEOL5410 apparatus. The shapes and sizes of the prepared nano-geopolymer were determined using Transmission electron microscopy (TEM) with micro-analyzer electron technique (JEOL JX 1230), while energy dispersive X-ray analysis (EDX) was determined using JEOL JX 2840 apparatus. The main functional group presence in the synthetic geopolymer was determined using Fourier-transform infrared spectroscopy (FT-IR). FT-IR spectrums were recorded on a Jasco FTIR- 6100 system, using a pellet made with dehydrated KBr and an instrument in the reflectance mode. The FTIR spectrums were determined between 400 to 4000 cm^-1^.

### Adsorption experiments

A stock solution of 1g/L was prepared by dissolving (1g) of MB in one liter of double distilled water. The working solutions were prepared by diluting the stock solution with double distilled water to give the required concentration of the working solutions. The hydrogen ion concentration (pH) of the solutions was adjusted using either 0.1N HCl or 0.1N NaOH solutions. The dye concentration was detected by Spectrophotometer (HACH DR 2800) at the maximum wavelength of the dye, λmax = 667 nm. The hydrogen ion concentrations were determined using XS Instrument pH meter. The optimum pH was firstly determined for pH ranging from 2 to 12.

Adsorption can be affected by various factors such as pH, initial concentration of adsorbates, contact time, adsorbent dosage, adsorbent size, and temperature, in addition to agitation speed. The optimization of the above-mentioned factors is essential to maximize outcomes of adsorption, percentage removal, or the adsorption capacity. The adsorption capacity is the predominant. The optimum pH was firstly determined for pH ranging from 2 to 12. The effect of initial conc, time, and temperature were examined according to the principle of Box-Behnken Design.

### Experimental design of adsorption of MB on nano-geopolymer

Box-Behnken design (BBD) is used to design and optimize the main parameters affecting the removal efficiency of the MB solution using Design-Expert version 13 software. Three variables were used in the design of the experiments: adsorption time (10–180 min), adsorption temperature (25–60  ℃), and initial concentration of the MB (20–300 mg/L) while keeping the adsorbent dose fixed during the experiments (0.1 g). The removal efficiency of the MB solution was used as a response value of the experimental design.

**Figure 2:**
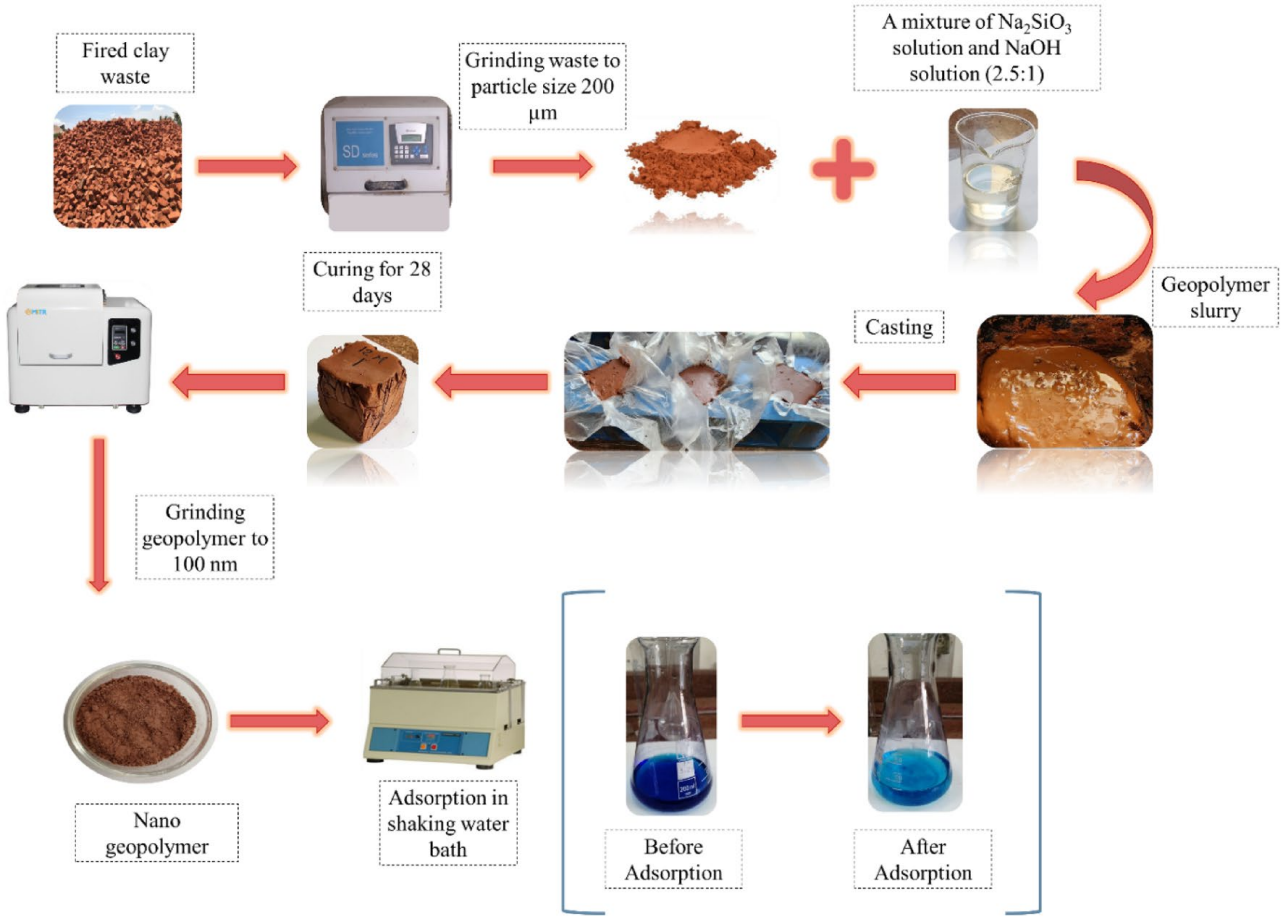
Experimental Procedure of MB Adsorption Using NHGP.

0.1 g of the synthetic nano-Homra-geopolymer (NHGP) was immersed in 100 mg/L of MB solution at room temperature followed by adjusting pH to 12 then placed in a water bath shaker with a speeding rate of 130 rpm. The experiment procedure is illustrated in Fig. 2. The effect of the initial concentration, time, and temperature were studied. Table 1 shows the conditions of the experimental design. The removal efficiency, R, and the adsorption capacity, q, were determined using the following Eqs. (1 and 2)^44^.

1 $$R \left(\%\right)=\frac{{C}_{0}-{C}_{e}}{{C}_{o}} x 100$$

2 $$q=\frac{\left({C}_{o}-{C}_{e}\right)x V}{m}$$

where ${C}_{0}$ is the initial concentration of MB solution in (mg/L), ${C}_{e}$ is the concentration at final time in (mg/L), $V$ is the volume of the solution sample (L), $m$ is the amount of the adsorbent in (g).

**Table 1:** Experimental Condition of Removal Efficiency of MB Using Design Expert.

Run	A: Time min	B: Temperature ℃	C: Initial Conc mg/L
1	95	60	300
2	10	42.5	300
3	95	60	20
4	10	25	160
5	95	42.5	160
6	95	42.5	160
7	180	42.5	300
8	10	42.5	20
9	95	42.5	160
10	180	60	160
11	95	42.5	160
12	180	42.5	20
13	180	25	160
14	95	25	20
15	95	25	300
16	95	42.5	160
17	10	60	160

### Adsorption isotherm, kinetics and thermodynamics of MB

The adsorption isotherm of the MB solution was determined at the optimum conditions using 0.1 g of nano geopolymer adsorbent at different initial concentrations ranging from 20 to 300 mg/L which mainly affects the adsorption capacity. Different adsorption models^45,46^ (Langmuir, Freundlich, Temkin, and Dubinin–Radushkevich) were used to identify the most fitted adsorption isotherm model. Langmuir adsorption isotherm assumes the absence of interactions between the adsorbed components, equal energy of sorption, and also homogenous binding sites^47^. Freundlich adsorption isotherm can describe the adsorption for both mono and multilayer^48^. Temkin adsorption isotherm assumes a linear equation and ignores the low and high concentrations. The uniform distribution of bounding energy was also assumed^45^. Dubinin–Radushkevich (Dub-Rad) isotherm model is used to determine the apparent free energy of the adsorption process and also the characteristic porosity^49–51^.

The adsorption isotherm model can be determined according to the following Eqs. (3) to (7):

3 $$Langmuir: \frac{1}{{q}_{e}}=\frac{1}{{q}_{m}}+\frac{1}{{q}_{m}{K}_{l}{C}_{e}}$$

4 $$Freundlich:log{q}_{e}={logK}_{f}+\frac{1}{n}log{C}_{e}$$

5 $$Temkin: {q}_{e}=B ln A+B ln{C}_{e}$$

6 $$Dub{-}Rad : q_{e} = q_{m} {\text{exp}}\left( { - \beta \varepsilon^{2} } \right)$$

7 $$\varepsilon =RTln(1+\frac{1}{{C}_{e}})$$

where ${C}_{e}$ is the concentration of MB solution at equilibrium in (mg.l^-1^); ${q}_{e}$ is the adsorption capacity at equilibrium in (mg.g^-1^); ${K}_{l}$ is the constant of Langmuir (l.mg^-1^); ${q}_{m}$ is the maximum theoretical adsorption capacity of the adsorbent in (mg.g^-1^); ${K}_{f}$ is the constant of Freundlich in (mg ^(1-n)^ .L^n^.g^-1^); finally $n$ is the adsorption strength constant for Freundlich. B is a Temkin constant (J.mol^-1^) related to the heat of adsorption and is equal to (RT/b), where T is the absolute temperature in (K) and R is a gas constant (8.314 J.mol^-1^.k^-1^) while Temkin isotherm constant is expressed by A (L.g^-1^). β is the Dub-Rad constant in (mol^2^/J^2^), while ε is the Polanyi potential that represents the theoretical adsorption capacity in (J/mol)^52^.

The kinetics of the adsorption process of MB at the optimum conditions using the Pseudo-first order, the Pseudo second order, the Intraparticle, Interparticle diffusion, and Elovich models. Equations (8) to (12) are used to investigate the kinetic model of the adsorption in the linear form^35,53–56^.

8 $$\text{Pseudo}-\text{first order}:ln\left({q}_{e}- {q}_{t}\right)=ln{q}_{e} - {k}_{1}t$$

9 $$Pesudo-second order: \frac{t}{{q}_{t}}= \frac{1}{{{k}_{2}q2}_{e}}+\frac{t}{{q}_{e}}$$

10 $$Intraparticle diffusion:{q}_{t}={k}_{p}{t}^{0.5}+c$$

11 $$ Interparticle diffusion: {ln}\left(1-{\left(\frac{{q}_{t}}{{q}_{e}}\right)}^{2}\right)=-{k}_{MD}.t$$

12 $$ Elovich:Q=\left(\frac{1}{\beta }\right){ln}\left(\alpha \beta \right)+\left(\frac{1}{\beta }\right)lnt$$

where *q*_*t*_ and *q*_*e*_ are the adsorption capacity at any time and equilibrium respectively in (mg/g), t is the time (min) and c is constant (mg.g^-1^). The rate constants for Pseudo-first order, Pseudo-second order, and Intraparticle diffusion are *k*_*1*_ (min^-1^), *k*_*2*_* (*g. mg^-1^. min^-1^) and *k*_*p*_ (mg.g^-1^.min^-0.5^) respectively. *k*_MD_ represents the mass transfer diffusion rate constant. α represents the initial absorption rate in (mg.g^-1^.min^-1^), while β is the Elovich constant.

Adsorption thermodynamics was used to identify the energy changes accompanied by the adsorption process. Different parameters were used to check the spontaneous of the adsorption process such as the change of Gibbs free energy (ΔG, kJ/mol), entropy change (ΔS, kJ/K), and enthalpy change (ΔH, kJ/mol)^24^. These parameters were calculated using the following equations from (13) to (15)^57,58^.

13 $${K}_{d}=\frac{{q}_{e}}{{C}_{e}}$$

14 $$ln{K}_{d}=\frac{\Delta {S}^{o}}{R}-\frac{\Delta {H}^{o}}{RT}$$

15 $$\Delta {G}^{o}=\Delta {H}^{o}-T{\Delta S}^{o}$$

where C_e_ is the concentration of the liquid phase at equilibrium in (mg/L), q_e_ is the adsorption capacity at equilibrium, R is the universal gas constant, K_d_ is the coefficient of distribution and T is the absolute temperature in Kelvin.

### Reusability test of nanogeopolymer

After obtaining the optimal removal efficiency of the adsorbent, a regeneration experiment utilizing a nano-geopolymer adsorbent was conducted. The ideal nano-geopolymer adsorbent, as determined by the experiment, was submerged in a certain pH solution at the optimum conditions. This allowed for three cycles of adsorption tests to ascertain the adsorption stability. After being filtered out, the nano-geopolymer adsorbent was washed five times with methanol (99%) before being dried one last time to remove any last traces of methanol. For every experiment, the MB dye's adsorption capacity and removal efficiency were computed.

## Results and discussion

### Characterization of adsorbent

#### Mineralogical analysis of nano-geopolymer adsorbent

X-ray Diffraction (XRD) is a technique used for determining the atomic and molecular structure of a crystalline material, in which the crystalline structure causes a beam of incident X-rays to diffract into many specific directions.

**Figure 3:**
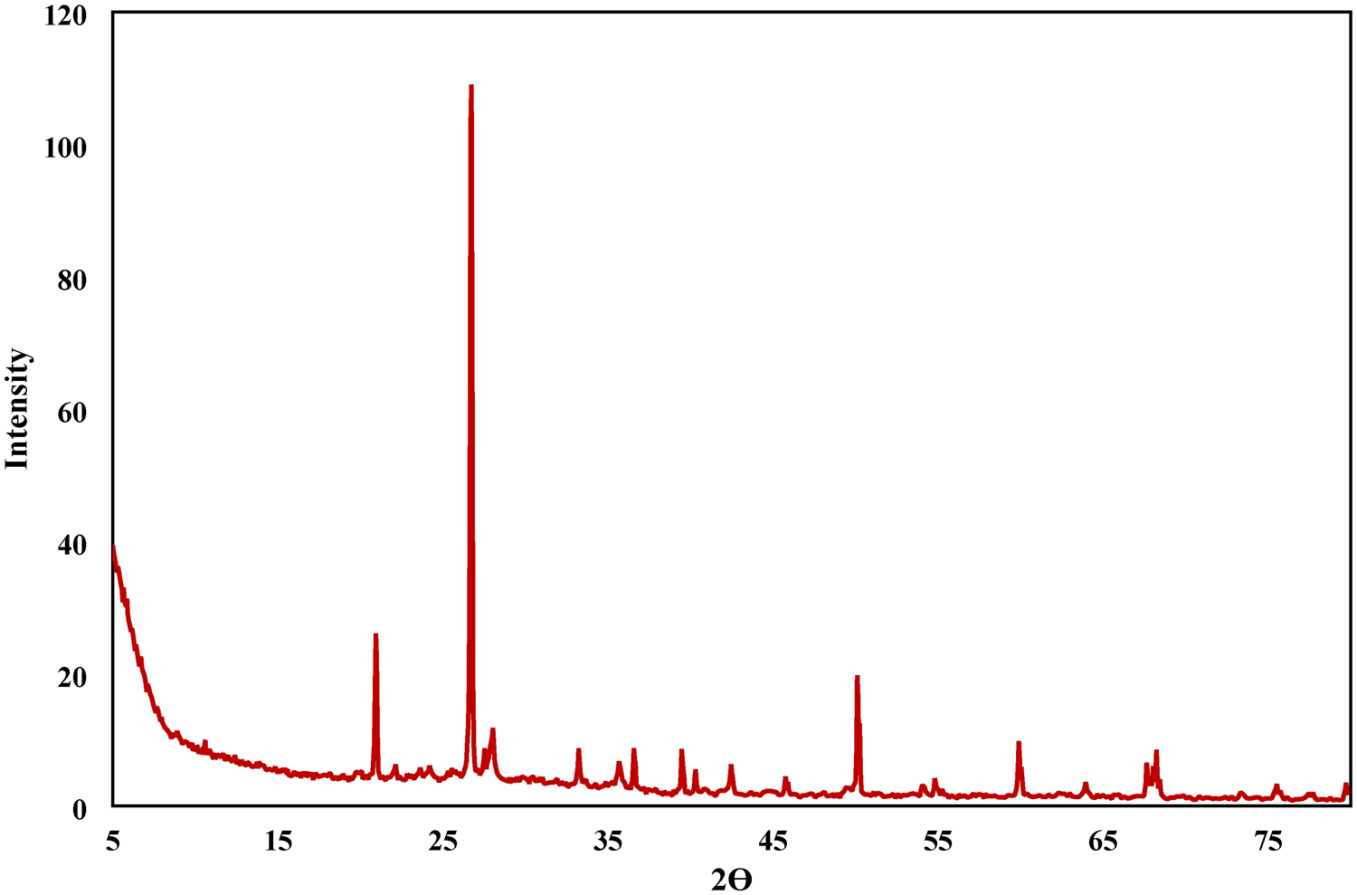
XRD of Adsorbent.

XRD has revealed that the main phase present in clay brick waste (Homra) is quartz at 2 ϴ equal to 20.936 and 26.7 as shown in Fig. 3. The firing of the bricks was done below 900 ℃, so the main constituents are quartz and amorphous meta-kaolin.

#### SEM of NHGP

**Figure 4:**
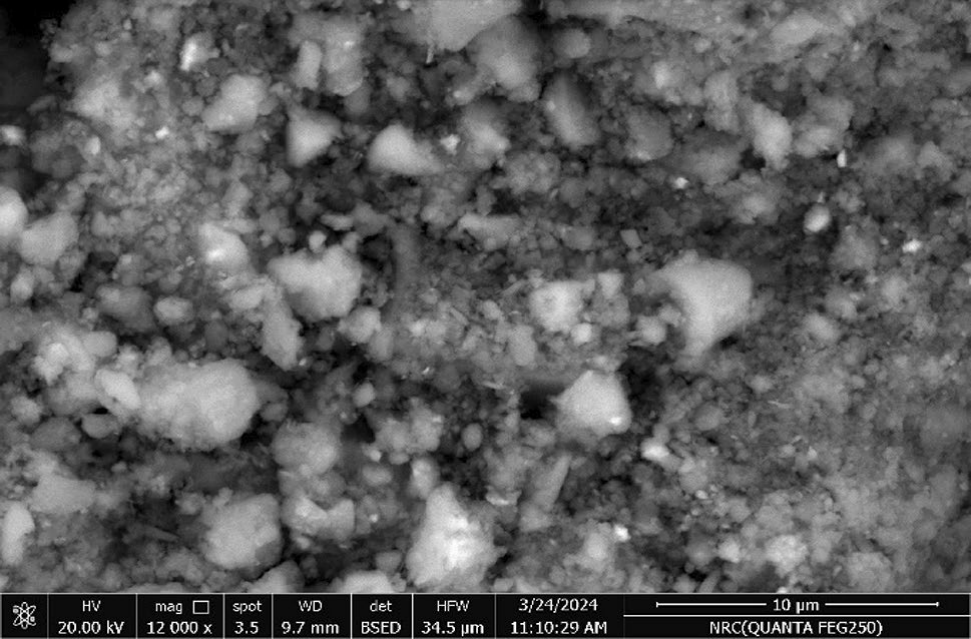
SEM of Homra Nano-geopolymer (NHGP).

To inspect the NHGP surface, SEM analysis was investigated. The SEM image with 12,000 × magnification depicted in Fig. 4 shows the formation of the heterogeneous matrix after the polycondensation of raw material. The geopolymer surface was consistent and coarse, with irregular aggregates, as seen in Figure. There were several tiny cavities and holes found with different shapes and sizes in the range of (12–44 nm). This suggests that the geopolymer may operate as an excellent adsorbent.

The surface structure of the NHGP is valuable because it gives superior vacancies for adsorbate molecules.

#### TEM analysis of NHGP

**Figure 5:**
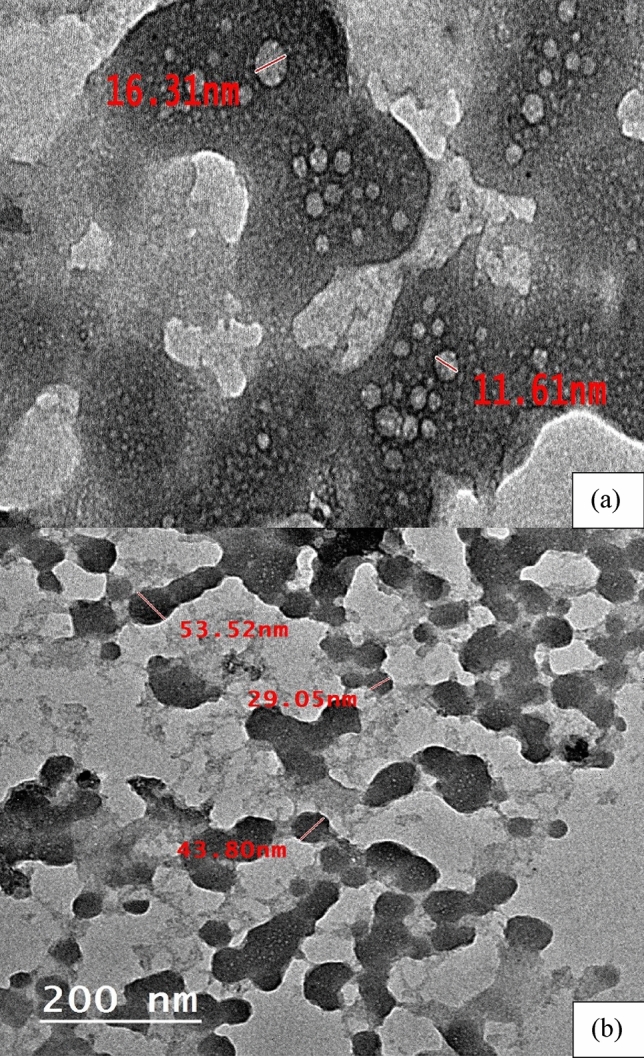
TEM of Homra Nano-geopolymer (NHGP): (**a**) 100 nm Magnification, (**b**) 200 nm Magnification.

Figure 5 shows an image of the prepared geopolymer matrix with Na_2_SiO_3_/ NaOH ratio 2.5 after 28 days of aging. There are many small particles with sizes ranging from 11.61 to 53.52 nm. These small particles are crystalline material as illustrated in Sect. (3.1.1).

It also indicates the presence of two different phases which are light and dark grains. The image of the dark grains is related to the presence of SiO_2_ in quartz form while the light-colored grains are related to Al_2_O_3_. The remaining different gray shade zones are related to aluminosilicates and a mixture of the most common phases. Aluminosilicates partially dissolve in the alkaline medium, producing an amorphous geopolymer gel along with undissolved particles of crystalline material during the reaction of geopolymerizatio^59^.

#### Analysis of specific area (BET)

The specific surface area of GP and NHGP were analyzed using BET and the results are illustrated in Table 2. It can be observed that the NHGP has a surface area of 18.36 m^2^/g more than that of GP 11.82 m^2^/g. The high specific surface area of NHGP makes it a good candidate to be used as an adsorbent medium. Generally, the adsorbent’s pores are classified into micropores, mesopores, and macropores with diameters less than 2 nm, ranging from 2 to 50 nm and more than 50 nm respectively according to IUPAC regulations^60^. The obtained results show that the produced adsorbent is mesopores type (as discussed in section "TEM analysis of NHGP") which is the most accessible pores to adsorb MB. The prepared geopolymer is an ideal adsorbent that is eco-friendly, non-soluble, mostly efficient, and easy preparation materials. Table 2 shows the BET analysis of the produced adsorbent and different adsorbent from previous studies.

**Table 2:** BET Analysis.

	Average pore reduction (nm)	Surface area BET (m^2^/g)	Pore volume (cm^3^/g)	Total pore volume (cm^3^/g)	References
Nano geopolymer	7.0738	18.3658	0.061697	6.496 × 10^–2^	Present study
Geopolymer	7.545	11.82	0.058	6.12 × 10^–2^	Present study
Kaolin, Metakaolin, Fly ash Geopolymer	9.68428	9.5076	0.02387	–	^9^
Gangue waste geopolymer	30.08	18.54	0.024	–	^61^
Amino-bagasse/metakaolin geopolymer	7.7604	49.16	0.1227	–	^62^
Steel slag, Fly ash, Metakaolin geopolymer	9.73	–	0.027	–	^63^

**Figure 6:**
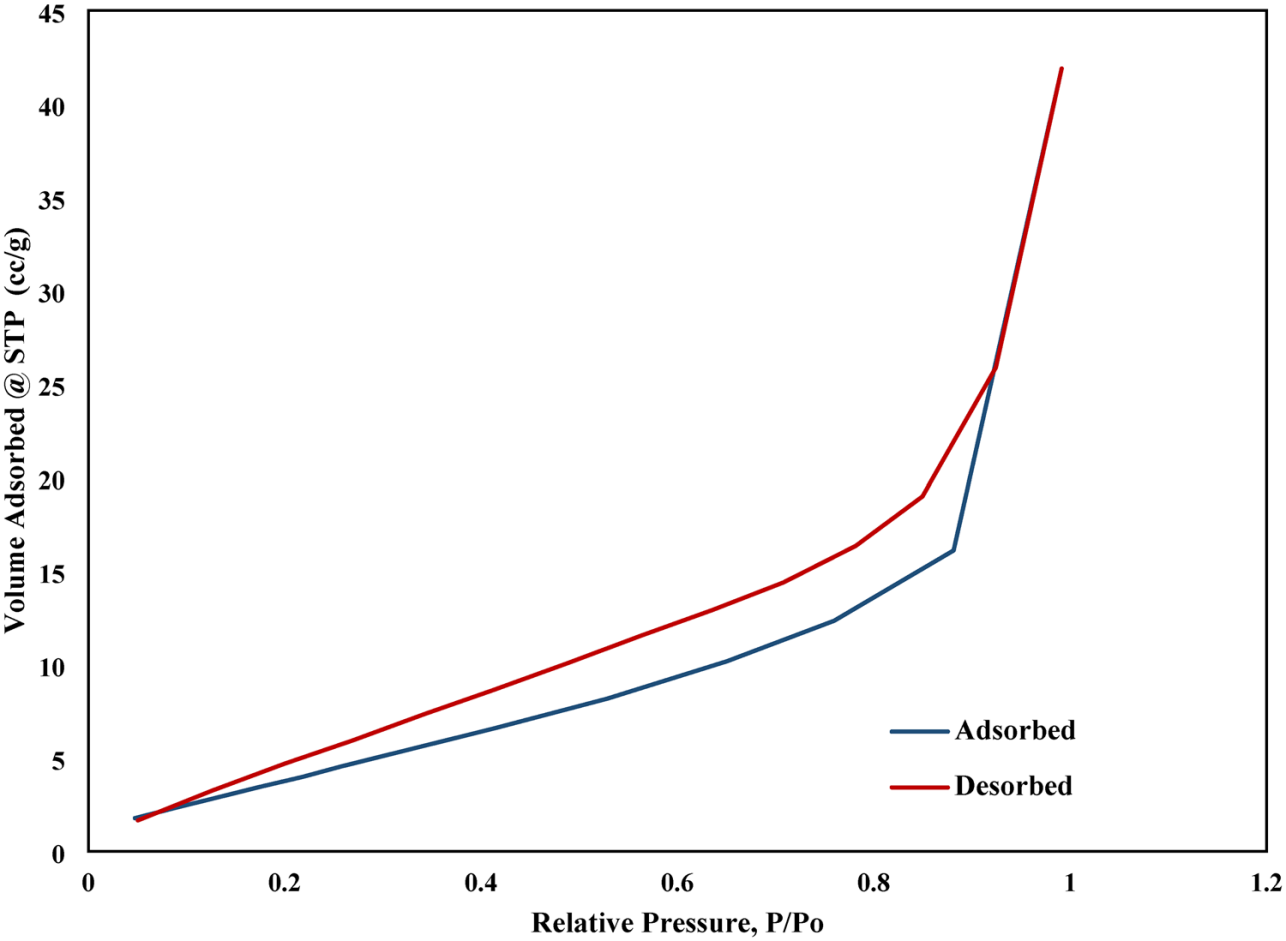
N_2_ Adsorption–Desorption Isotherms of N_2_ onto NHGP.

From Fig. 6, it was observed that lower pressure regions show the formation of a monolayer followed by a formation of multilayers. This model is Type IV isotherms with hysteresis loop at a relative pressure till 0.9. BET surface area characterization of NHGP is mesoporous materials, with pore diameters ranging between 2—50 nm giving this type of isotherm.

#### EDX and FTIR analyses

**Figure 7:**
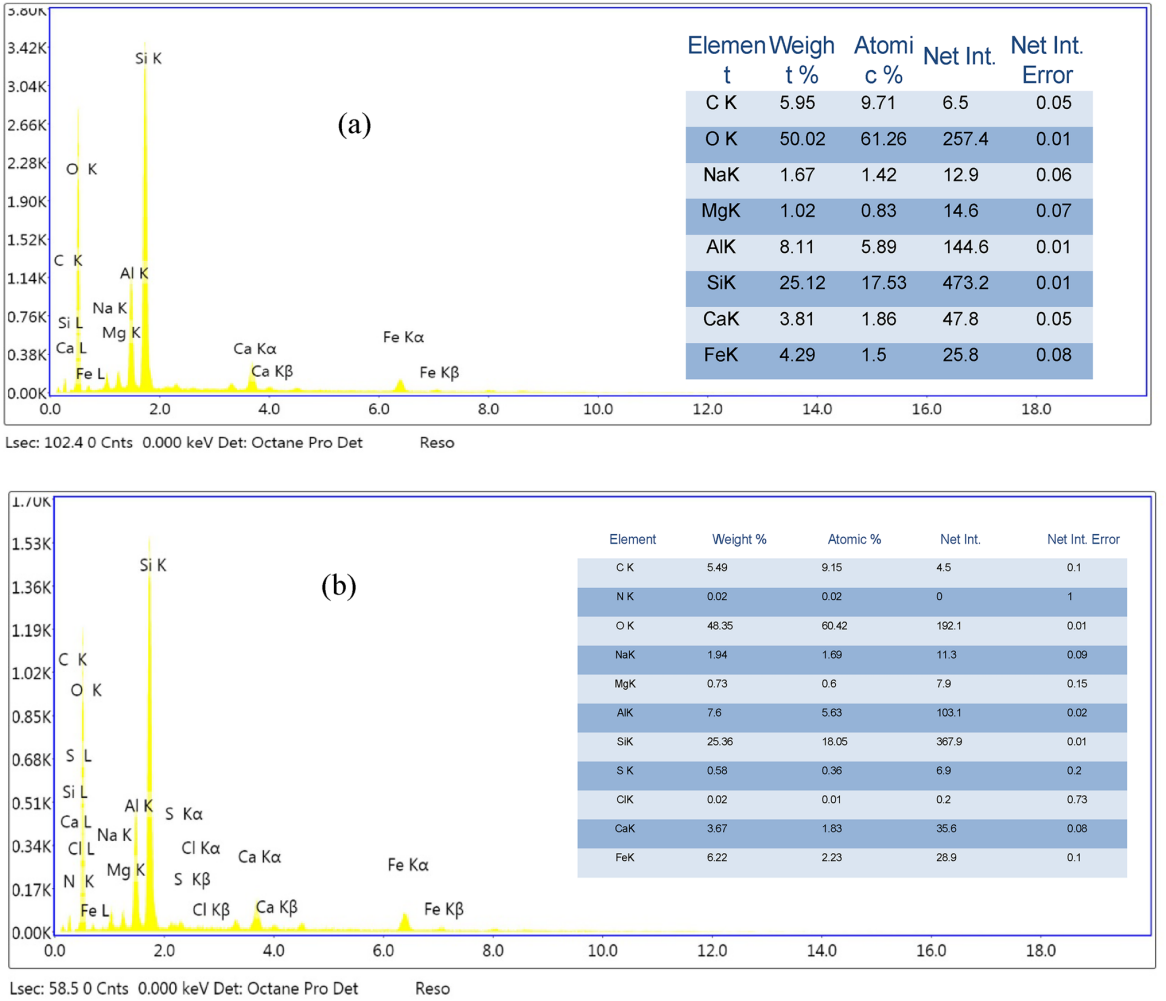
EDX Analysis (**a**) before adsorption, (**b**) after adsorption of MB.

The chemical analysis of NHGP (EDX analysis) depicted in Fig. 7a contains 25.12 wt% Si, 8.11 wt% Al, and 50.02 wt% O, and there is no sulfur, Cl, or N content in the analysis of NHGP as native adsorbent. It illustrates the composition ratio of Si and Al to be 3.1.

Compared with NHGP after the adsorption of Methylene blue dye chemical analysis, Fig. 7b, which contains mainly 25.36 wt % Si, 7.6 wt% Al, 48.35 wt% O, with the presence of S, Cl, and N which are compatible with the adsorption of MB.

**Figure 8:**
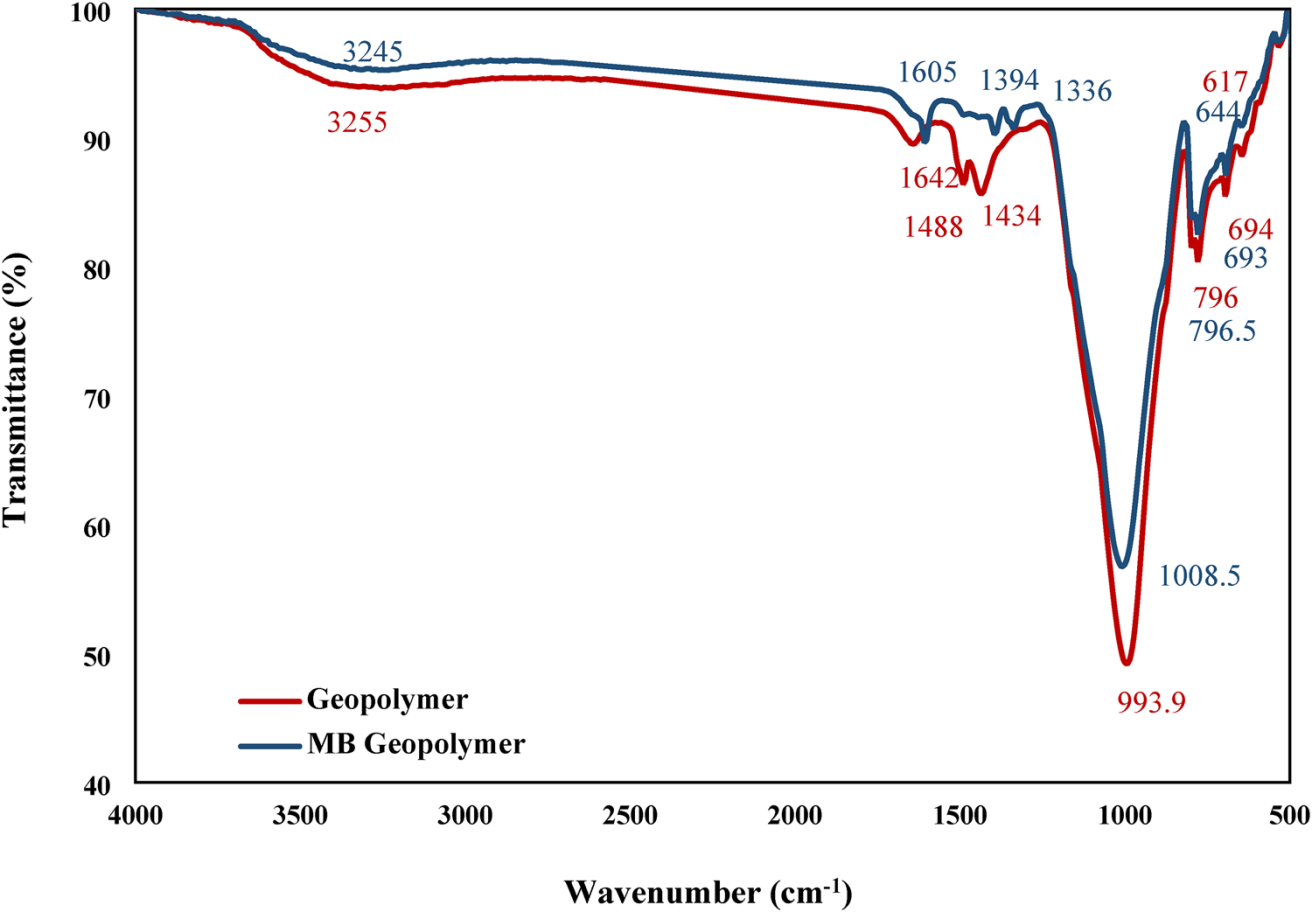
FTIR Analysis of Geopolymer before and after Adsorption.

FTIR was used to identify the functional groups involved in the adsorption of MB before and after the adsorption process onto NHGP as shown in Fig. 8. A strong vibration peak centered at 993 cm^-1^ typical for aluminosilicates can be seen. This peak shifts to 1008 cm^-1^ with the adsorption of MB. Al–O–Si vibrations correspond to the absorption bands 600–800 cm^-1^. The absorption peak of 796 cm^-1^ was an indication of the presence of quartz^64^. The absorption band around 1443 cm^-1^ is attributed to stretching vibrations of CO_3_^2-^ ions confirming the existence of carbonate species^65^. The broad band observed at 3255 cm^-1^ and for geopolymer, is due to the OH and the adsorbed water this peak shifted to 3245 on the adsorption of MB. The stretching bands at 1008.5 cm^-1^ are for (-NH_2_) and 1390 cm^-1^ for (-CH_3_) that are present in MB that adsorbed onto geopolymer. The peak at 1605 cm^-1^ is due to asymmetric stretching vibrations of C = O and the peak observed at 1394 cm^-1^ can be assigned to the aromatic compound group^66^.

### Effect of pH on MB adsorption

**Figure 9:**
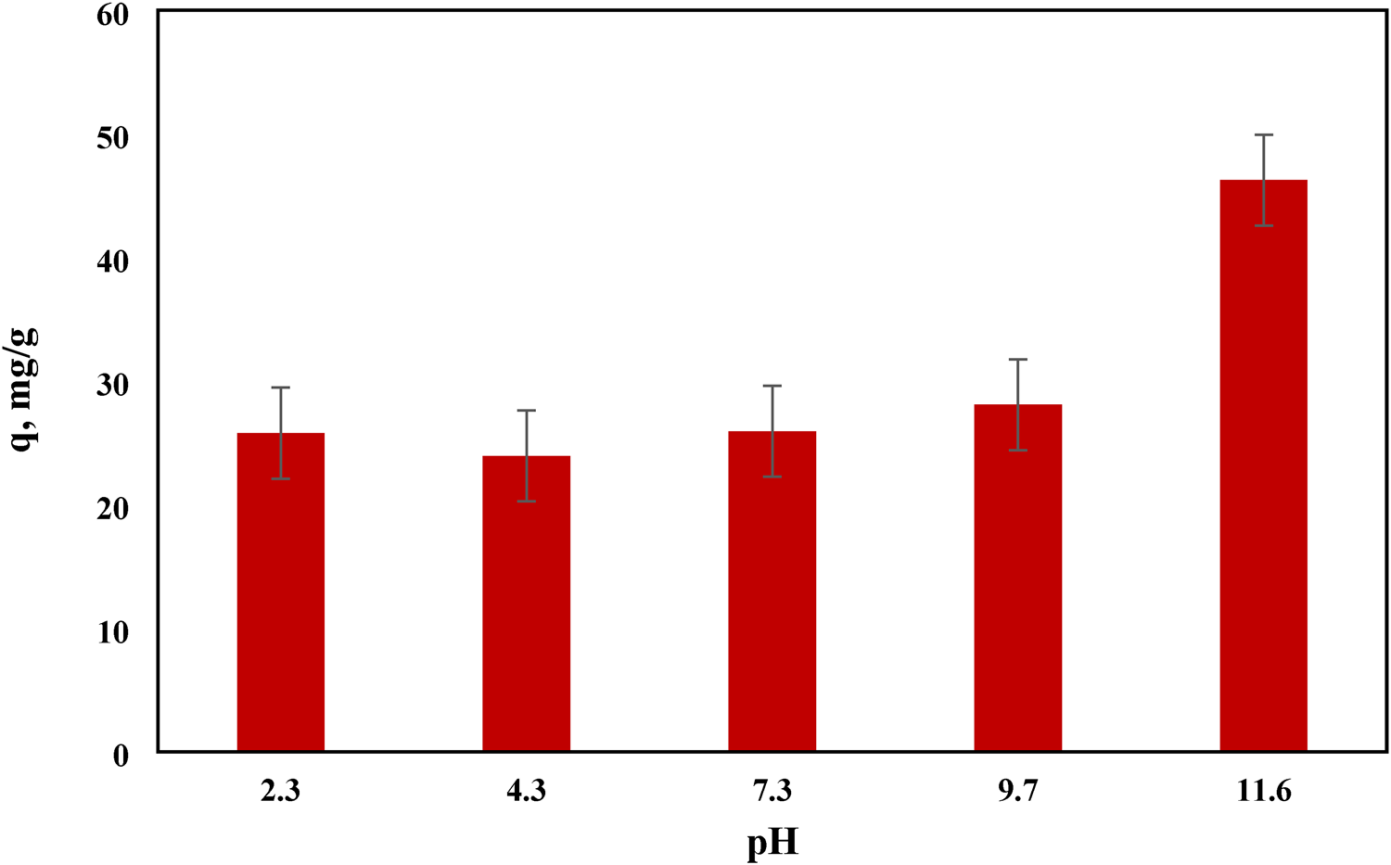
Influence of pH on the Adsorption Capacity of MB Dye onto NHGP.

The adsorption of methylene blue (MB) using NHGP at different pH values is presented in Fig. 9. As demonstrated from the figure, the quantities of MB adsorbed onto the resin were largely raised with the increase in pH, designating that the alkaline environment was more advantageous for MB adsorption. Bearing in mind the cationic nature of MB, electrostatic interactions may occur between MB and the adsorbent surface^67^^,^^68^.

**Figure 10:**
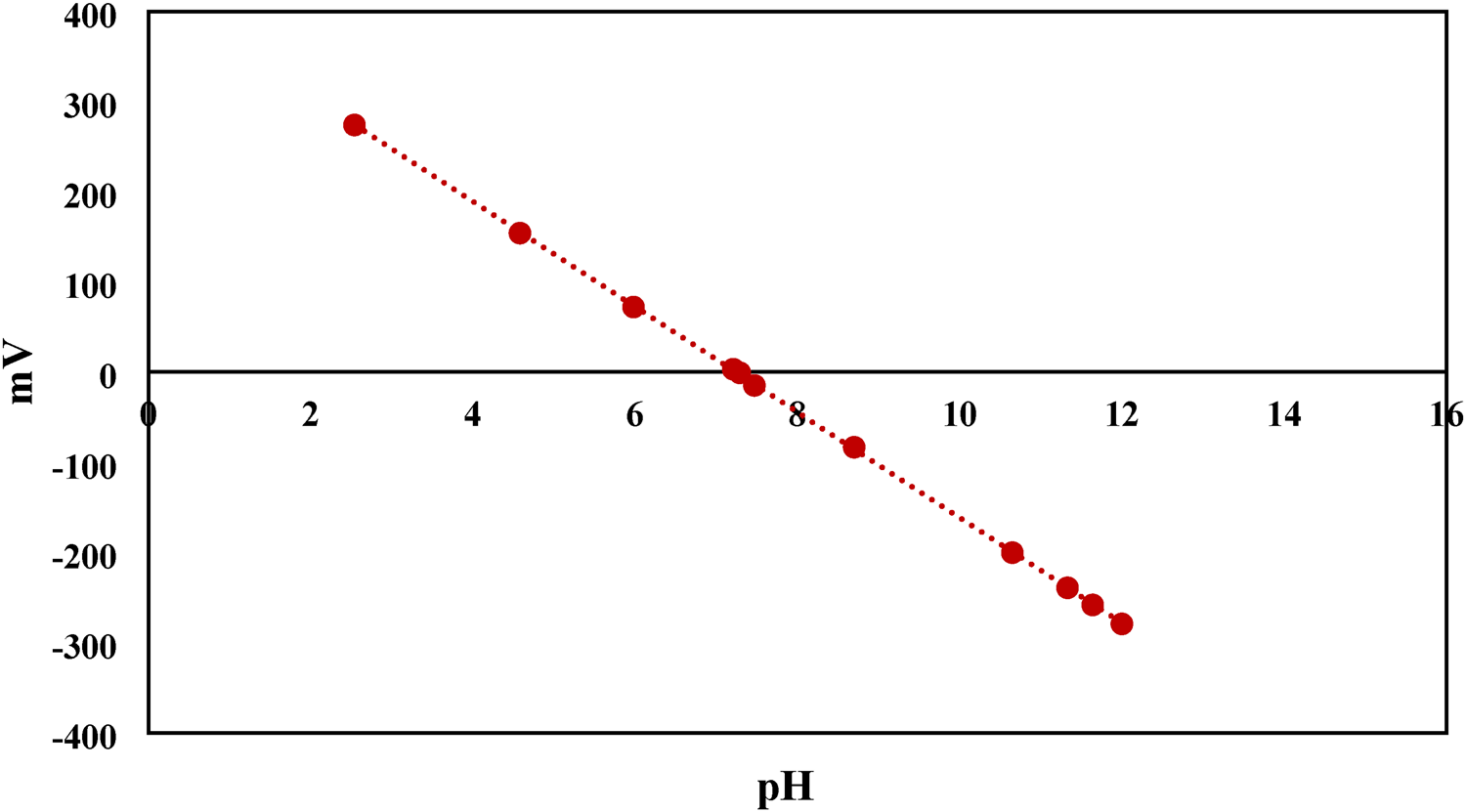
Zeta Potential of NHGP at Different pH.

**Figure 11:**
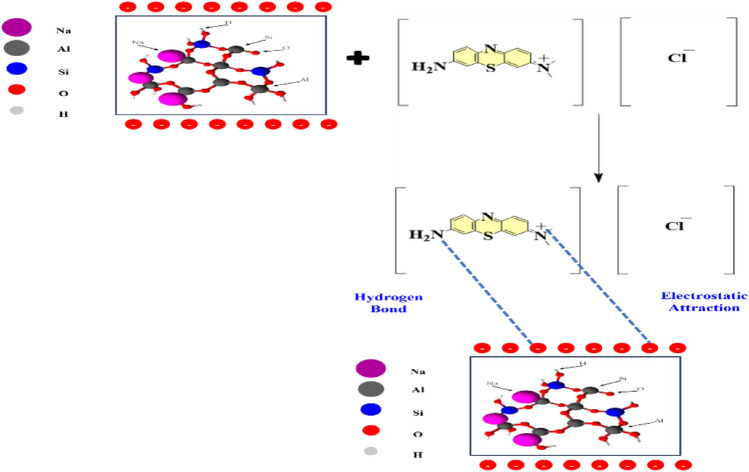
Schematic Diagram for the Proposed Reaction between MB and NHGP.

Figure 10 illustrates the zeta potential of the geopolymer at different pH’s. As a consequence of this data, the zeta potential is negative at the alkaline medium, therefore the surface of NHGP was negatively charged. Thus, electrostatic attraction between MB cations and the adsorbent surface was expected higher pH values. The adsorption of MB onto NHGP is not dependent only on the Electrostatic interactions, as there are some adsorptions that occur at low pH values (Fig. 10). Another mechanism as well as hydrophobic adsorption and electron donor–acceptor (EDA) interactions may affect the adsorption process, this proposed interaction can be seen in Fig. 11^69^–^71^.

### ANOVA of MB removal

Table 3 lists the actual and the predicted values of the removal efficiency of MB that was performed according to the designed experiments using Design-Expert v13. The relationship between the different parameters and the removal efficiency of MB was described using multivariate analysis. It was found that the quadratic model was the best fitness function that describes this relation as following Eq. (16):

16 $$Y=48.61731+ 0.144115 t-0.493188 T -0.298732 C + 0.007188 tT + 0.000346 tC + 0.005838 TC -0.001421 {t}^{2} -0.000306 {T}^{2} -0.000068 {C}^{2}$$

where Y is the removal efficiency of MB, t is the time of the adsorption, T is the temperature and C is the initial concentration. According to the ANOVA analysis in Table 4, the model is significant and the p-value of lack of fit is 0.3895 which is non-significant (more than 0.05).

**Table 3:** Experimental Conditions of Actual and Predicted Values of Removal Efficiency of MB from Wastewater.

Run	A: Time min	B: Temperature ℃	C: Initial conc mg/L	Actual removal efficiency MB (%)	Predict removal efficiency MB (%)
1	95	60	300	81.6	78.99
2	10	42.5	300	9.6	11.21
3	95	60	20	60.15	61.42
4	10	25	160	13.9	13.56
5	95	42.5	160	52.78	52.42
6	95	42.5	160	50.25	52.42
7	180	42.5	300	57.13	59.40
8	10	42.5	20	32.76	30.49
9	95	42.5	160	52.8	52.42
10	180	60	160	91.6	91.93
11	95	42.5	160	56.97	52.42
12	180	42.5	20	63.8	62.19
13	180	25	160	33.13	32.13
14	95	25	20	49	51.61
15	95	25	300	13.24	11.97
16	95	42.5	160	49.3	52.42
17	10	60	160	29.6	30.60

**Table 4:** ANOVA Analysis of the Removal Efficiency of MB from Wastewater.

Source	Sum of squares	df	Mean Square	F-value	*p*-value	
Model	8192.33	9	910.26	91.06	< 0.0001	Significant
A-Time	3192.01	1	3192.01	319.30	< 0.0001	
B-Temp	2952.19	1	2952.19	295.31	< 0.0001	
C-Initial Conc	243.54	1	243.54	24.36	0.0017	
AB	457.32	1	457.32	45.75	0.0003	
AC	67.98	1	67.98	6.80	0.0350	
BC	818.25	1	818.25	81.85	< 0.0001	
A^2^	443.99	1	443.99	44.41	0.0003	
B^2^	0.0370	1	0.0370	0.0037	0.9532	
C^2^	7.43	1	7.43	0.7436	0.4171	
Residual	69.98	7	10.00			
Lack of Fit	34.56	3	11.52	1.30	0.3895	not significant
Pure Error	35.42	4	8.85			
Cor Total	8262.31	16				

The values of R^2^ and R^2^_adj_ are proportional rises with the significance of the model. Table 5 shows the coefficient of determination of R^2^ and R^2^_adj_ equal 0.9915 and 0.9805 respectively for the removal efficiency of MB, while the predicted value of R^2^ is 0.9264. Those values show a good agreement between the actual and predicted values presented by the model.

**Table 5:** Statistics fit model.

Std. dev	3.16	R^2^	0.9915
Mean	46.92	Adjusted R^2^	0.9806
C.V. %	6.74	Predicted R^2^	0.9264
		Adeq Precision	33.2902

Table 6 shows that all adsorption parameters affect the adsorption performance and removal efficiency of MB. The positive signs for parameters such as time, temperature, and the interaction between time and temperature have a great effect on the adsorption performance, while the negative signs for parameters such as initial concentration and the time square imply that increasing those parameters causes decreasing the performance of the adsorption.

**Table 6:** Values of VIF.

Factor	Coefficient estimate	df	Standard error	95% CI Low	95% CI high	VIF
Intercept	52.42	1	1.41	49.08	55.76	
t	19.98	1	1.12	17.33	22.62	1.0000
T	19.21	1	1.12	16.57	21.85	1.0000
C	− 5.52	1	1.12	− 8.16	− 2.87	1.0000
t T	10.69	1	1.58	6.95	14.43	1.0000
t C	4.12	1	1.58	0.3843	7.86	1.0000
TC	14.30	1	1.58	10.56	18.04	1.0000
t^2^	− 10.27	1	1.54	− 13.91	− 6.63	1.01
T^2^	− 0.0938	1	1.54	− 3.74	3.55	1.01
C^2^	− 1.33	1	1.54	− 4.97	2.31	1.01

**Figure 12:**
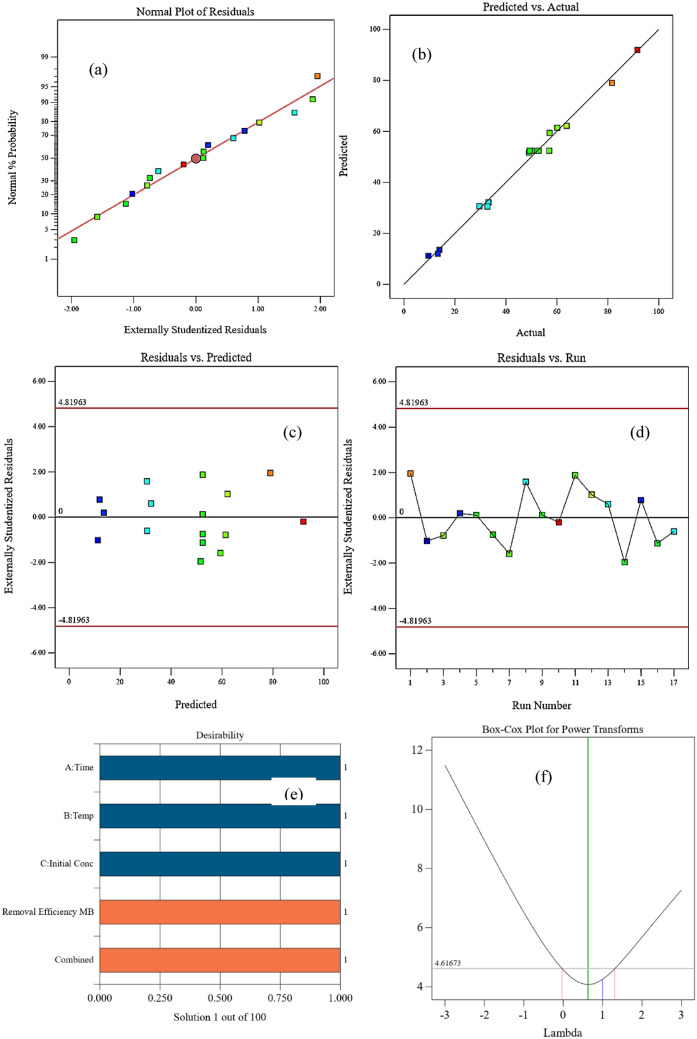
Diagnostic Plots for the Adsorption Removal Efficiency of MB: (**a**) Normal Probability, (**b**) Predicted and Actual Values, (**c**) Externally Studentized Residuals versus Predicted Values, (**d**) Externally Studentized Residuals versus Run Number, (**e**) Pareto Diagram, (**f**) Box-Cox Diagram.

The main diagnostic plots were observed in Fig. 12. The residual of the function model shows a good distribution probability that confirms the assumption accuracy and the independence of the residuals as shown in Fig. 12a. A linear correlation was observed between the predicted and actual values of the experimental data as shown in Fig. 12b while Fig. 12c shows a random distribution of the data points obtained from the experiments with the error line, it was indicated a good prediction and model accuracy. It was observed that the different data of the residuals are in the interval ranges of (4.81963) and (-4.81963) indicating a good prediction of the model as illustrated in Fig. 11d. Figure 12e shows the Pareto graph for the adsorption process that indicates the satisfaction of each variable to the criteria, their values near 1 represent the maximum adsorption reached. The best lambda for the adsorption process was found at 0.63 as illustrated in Fig. 12f the Box-Cox diagram which indicates that the data was suitable and didn’t need any improvement^44^^,^^50^.

#### Effect of interactive different parameters on adsorption removal efficiency of MB

**Figure 13:**
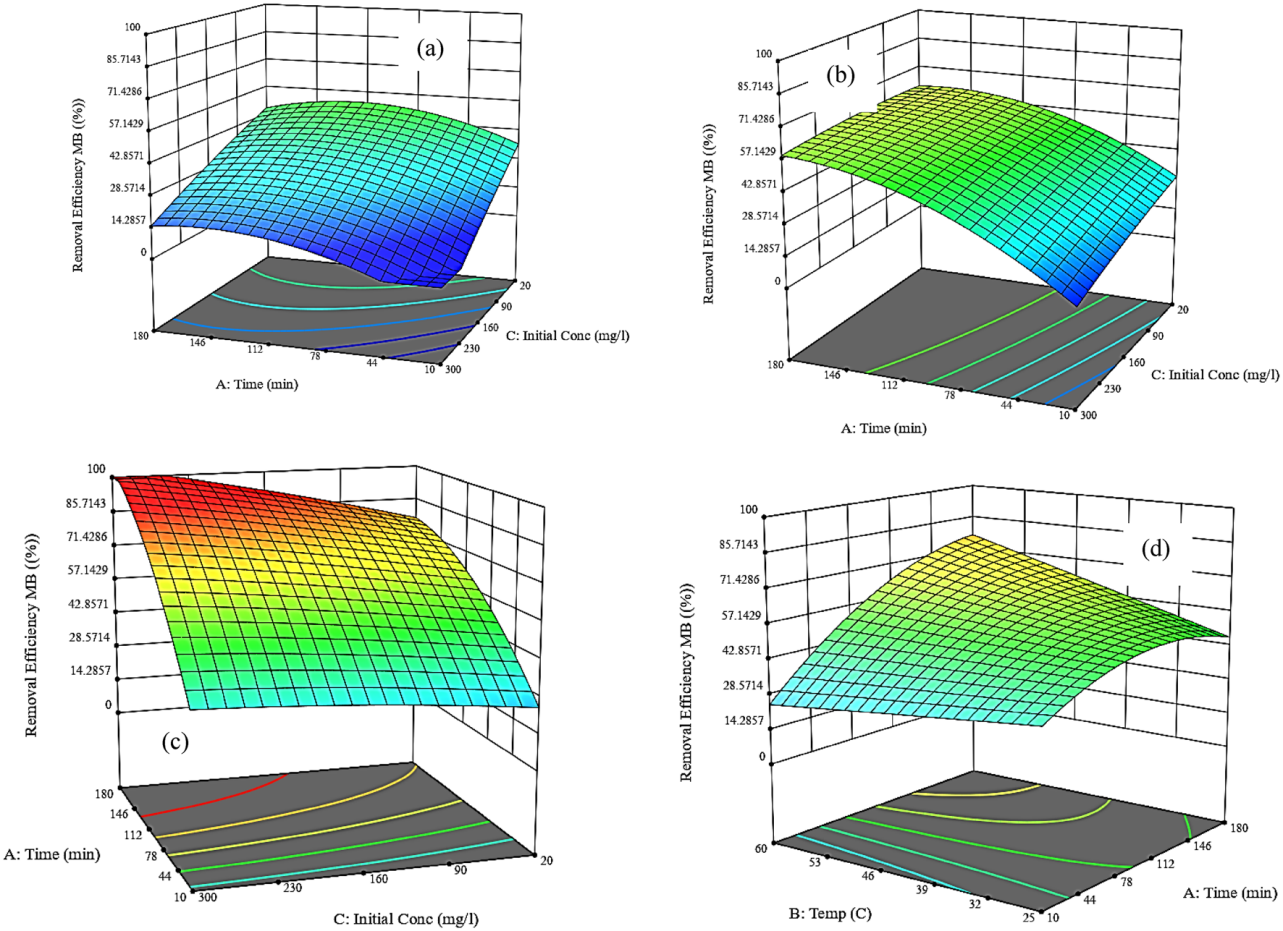

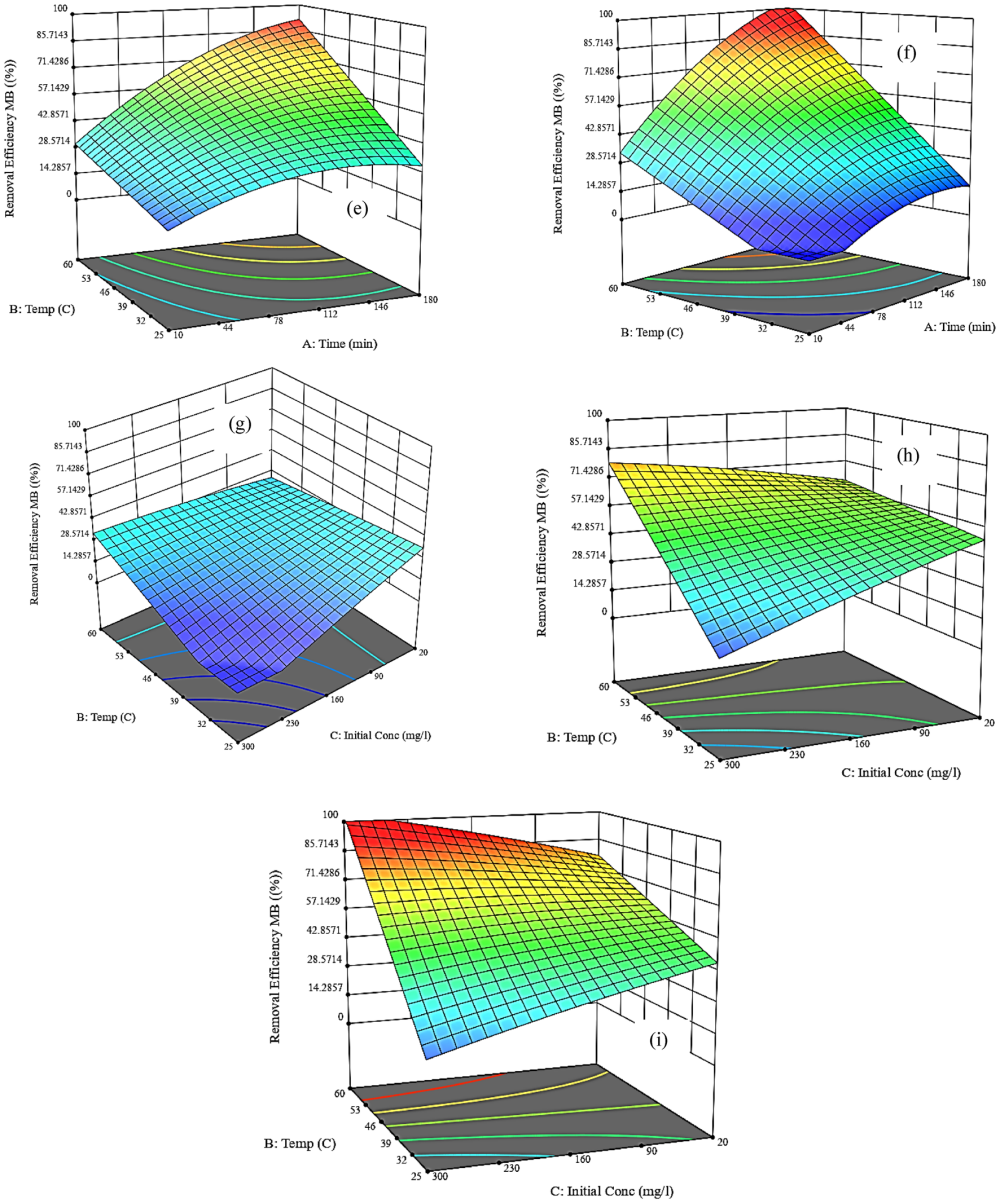
3D Response Surface Plots of for the Effect of Different Parameters on the Adsorption Removal Efficiency of MB.

Figure 13a–c show the relationship between the time, initial concentrations of MB at different adsorption temperatures (25 ℃, 42.5 ℃, and 60 ℃), and the adsorption removal efficiency of MB. It can be observed that increasing the temperature causes increasing in the removal efficiency of MB at different initial concentrations and times. Temperature affects the adsorption process based on the type of bonds formed between adsorbate sites and adsorbents. This behavior is due to the rapid diffusion of the MB dye molecules from the synthetic wastewater to reach the active sites present in the NHGP^72^ which indicates that a higher temperature is preferred for the adsorption process.

Figure 13d–f show the effect of contact time and adsorption temperatures at initial concentrations ranging from 20 to 300 ppm on the removal efficiency of MB. It can be understood that the effect of the adsorption time is more dominant than the effect of the adsorption temperature. It can be observed that the maximum removal efficiency of MB at a temperature of 60 ℃, a time of 180 min ranging from 80 to 100% at initial concentrations of 20 ppm and 300 ppm respectively. The reason for this behavior is that the high initial concentration of MB causes a high concentration gradient leading to the high driving force for the transfer of the molecules quickly to the active sites^73^. It was observed that increasing the initial concentration of MB causes increasing the loading capacity of NHGP adsorbent that complies with the results obtained from the adsorption of MB using green olive stones^66^.

Finally, Fig. 13g–i show the relationship between the adsorption temperature and initial concentrations at different contact times ranging from 10 to 180 min on the removal efficiency. It can be observed that the effect of the adsorption temperature is more dominant than the initial concentration and also increasing the contact time between the adsorbent and the MB solution causes increasing in the removal efficiency due to allowing a sufficient time to adsorb MB on the NHGP. Adsorption capacity increases as the contact time increases to a point, after which further increase in the contact time does not increase adsorbate uptake on adsorbent due to saturation and there will be no significant change in the removal efficiency of MB^74^.

#### Optimization of MB removal efficiency

The optimum removal efficiency of MB was obtained from the program software using the optimization tab. The constraints of each parameter were set as illustrated in Table 7. The optimal parameters were found to be at a temperature of 59 ℃, initial concentration of MB of 254 ppm, and adsorption time of 163.2 min to obtain a predicted value for removal efficiency of MB of 95% with the desirability of 1 as illustrated in Table 8. To validate these results the suggested optimum parameters were performed five times at the optimum conditions to calculate the mean removal efficiency of MB of 94.4% with a standard deviation of 1.4% with an error percentage of 0.63 which proves the validity of the model.

**Table 7:** Constraints Optimization.

Name	Goal	Lower limit	Upper limit	Lower weight	Upper weight	Importance
A: Time	is in range	10	180	1	1	3
B: Temp	is in range	25	60	1	1	3
C: Initial Conc	is in range	20	300	1	1	3
Removal Efficiency MB	maximize	9.6	91.6	1	1	5

**Table 8:** Results of the Optimum Solutions.

Number	Time	Temp	Initial conc	Removal efficiency MB	Desirability	
1	163.23	59.081	254.168	95.116	1	Selected
2	175.299	58.074	237.562	93.882	1	
3	167.275	58.437	223.843	92.423	1	
4	158.502	58.587	230.888	91.617	1	
5	179.406	57.34	222.832	91.893	1	
6	135.101	59.820	297.520	92.584	1	
7	154.257	59.565	229.569	92.556	1	

### Adsorption isotherm, kinetics and thermodynamics studies

#### Isotherm models of the adsorption of MB onto NHGP

**Figure 14:**
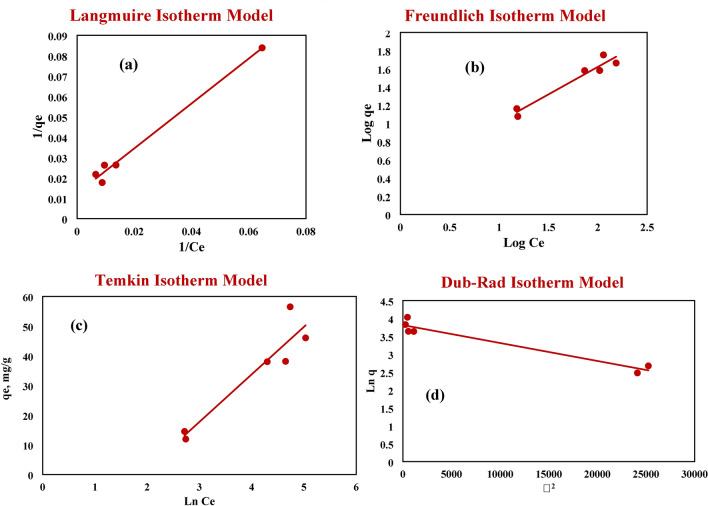
Adsorption Isotherm for MB onto NHGP; (**a**) Langmuir, (**b**) Freundlich, (**c**) Temkin.

Studying the Langmuir isotherm model, it has been stated that the adsorbent surface is homogeneously distributed having identical adsorbent sites and there is no lateral interaction forming a monolayer. From Fig. 14a it was found that the correlation coefficient of the model (R^2^) is equal to 0.988 which indicates that the adsorption of MB with NHGP is well fitted with this model with a maximum capacity of 80.65 mg/g.

Figure 14b represents the fitting of the isotherm data with the linear form of the Freundlish model. The Freundlich adsorption isotherm model is used to describe heterogeneous systems. From the fitting data and the correlation factor (R^2^ = 0.9418), we deduced that this system is moderately fitted with the Freundlish model.

The 1/n value obtained from the Freundlich isotherm plot is 0.6036 which falls between 0 and 1 indicating the adsorption is linear and uniform throughout the surface. According to the 1/n value (0 < 1/n < 1), this isotherm is a favorable physical process.

The Temkin model in Fig. 14c clarifies the variation in the heat of adsorption and the interactions of adsorbate–adsorbate molecules during the progression of the adsorbed layer. The correlation coefficient of this model is 0.8846 which indicates poor fitting for the adsorption of MB with NHGP. Figure 14d shows the Dub-Rad model that is suitable for moderate concentration of the adsorbate and assumes multilayer properties as Vander Waals forces are involved and the physical adsorption process can be applied^75^. Table 9 shows the fitting parameter of the four adsorption isotherm models.

**Table 9:** Isotherm Models Parameters of the Removal of MB by NHGP.

Model	Parameters	Values
Langmuir	q_m_ (mg/g)	80.65
	k_L_ (L/mg)	0.9077
	R^2^	0.9882
	E_p_	0.05–0.0037
Freundlich	K_f_, (mg/g) (mg/L)^(1/n)^	2.589
	n^-1^	0.6036
	R^2^	0.9418
Temkin	A (L/g)	0.151
	B (J/mol)	16.003
	R^2^	0.8846
Dub–Rad	q_m_ (mg/g)	45.388
	β (mol^2^/J^2^)	5.01153E-05
	R^2^	0.9383

#### Kinetics of the adsorption of MB onto NHGP

**Figure 15:**
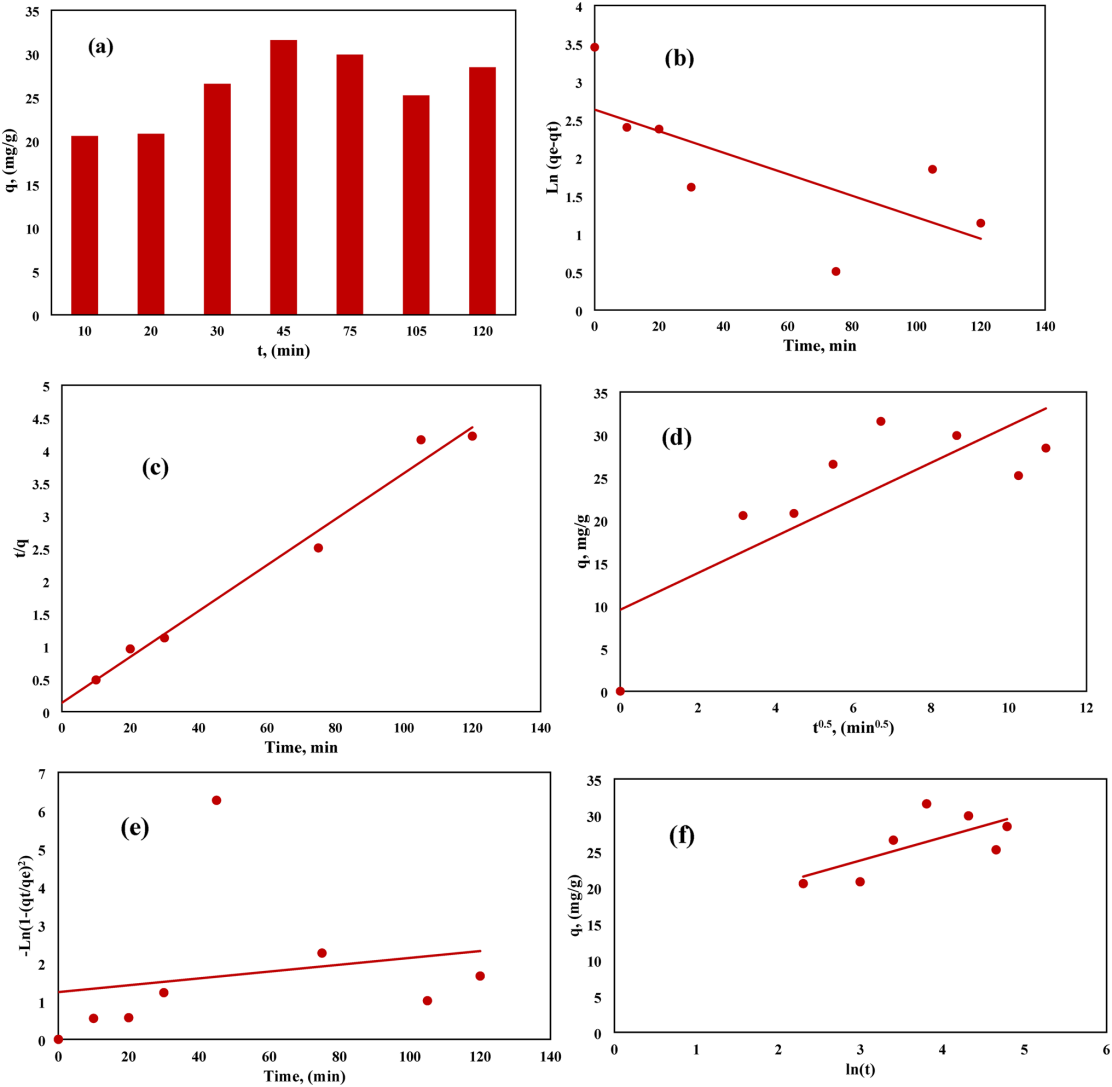
Kinetics of MB Adsorption; (**a**) Adsorption Capacity of MB versus Contact Time, (**b**) Pesudo First Order, (**c**) Pesudo Second Order, (**d**) Intraparticle Diffusion, (**e**) Interparticle Diffusion, (**f**) Elovich Model.

Figure 15a illustrates the adsorption capacity and the rate of the adsorption of MB onto the NHGP. It can be observed there is rapid adsorption takes place at a time ranging from 0 to 45 min to increase the adsorption capacity from 0 mg/g to 31.57 mg/g respectively. The removal efficiency of MB is enhanced as the contact time increases till reaches 45 min, then at times ranging from 45 to 120 min the adsorption capacity was nearly unchanged ranging from 31.57 mg/g to 28.44 mg/g due to reaching equilibrium.

Kinetics of MB adsorption on NHGP were determined using Pseudo first order, Pseudo second order, Intraparticle diffusion, Interparticle diffusion, and Elovich models as illustrated in Fig. 15b–f to identify the best-fitted model and analyze the MB adsorption process. Table 10 shows the fitting parameters for the five kinetic adsorption models, which designated that the Pseudo Second Order kinetic model is the most applicable model for this process. It has a correlation coefficient of (R^2^ = 0.9838) which is higher than the four other models. According to this model, it was found that the equilibrium adsorption capacity and adsorption rate constant were 28.41 mg. g^-1^ and 9.14 × 10^–3^ g. mg^-1^.min^-1^ respectively. Choosing of Pseudo Second Order model is considered the most suitable model for the adsorption process. It is also indicated that the adsorption is a chemisorption type and the adsorption rate doesn’t depend on the adsorbate concentration but depends on the adsorption capacity^76^ confirmed by the results obtained from the Design-Expert software.

**Table 10:** Kinetics Parameters of MB Adsorption onto NHGP.

Kinetics model	Parameters	NHGP Adsorbent
Pesudo First Order	k_1_ (min^-1^)	1.41 × 10^–2^
	q_e_ (mg/g)	31.57
	R^2^	0.57075
Pesudo Second Order	k_2_ (g. mg^-1^.min^-1^)	9.15 × 10^–3^
	q_e_ (mg/g)	28.41
	R^2^	0.9838
Intraparticle Diffusion	k_p_ (mg.g^-1^.min^-0.5^)	2.1499
	C (mg.g^-1^)	9.529
	R^2^	0.6301
Interparticle Diffusion	k_MD_	0.009
	R^2^	0.041
Elovich	β	0.313
	α	270.106
	R^2^	0.4647

#### Adsorption thermodynamics

**Figure 16:**
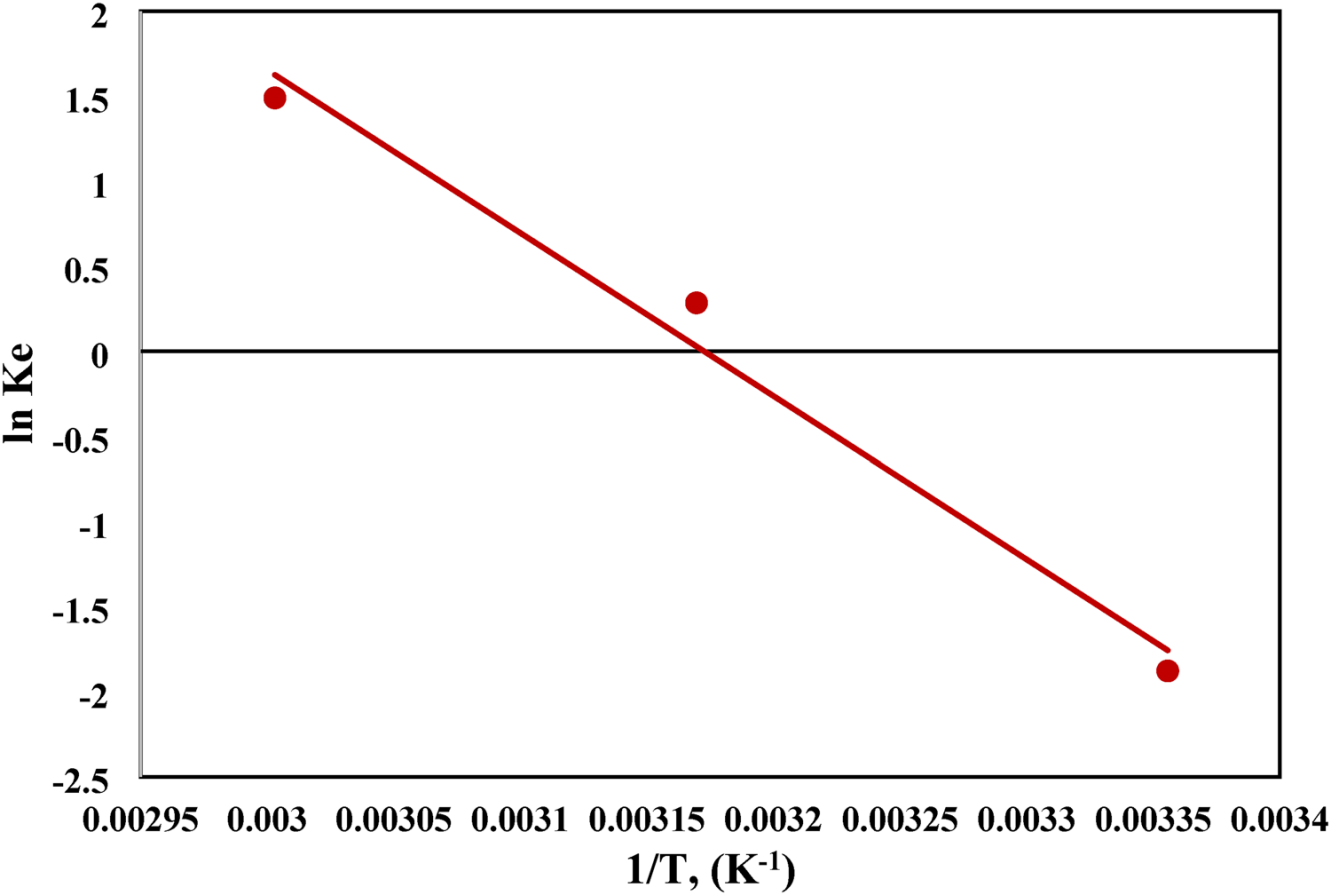
Linear Relationship of Van’t Hoff Equation at Different Adsorption Temperatures.

Thermodynamic parameters were assessed for the removal of MB dye by using NHGP synthesized adsorbent in the temperature range of 25 to 60 ℃. Figure 16 illustrates the calculations of the thermodynamics variables ΔS° and ΔH°. The K_d_ values were obtained from the adsorption data at different temperatures. The ΔG° values shown in Table 11 confirm that the adsorption process was spontaneous at the selected temperature range (25–60 ℃), which is in concurrence with the ΔS° value (ΔS > 0 for the spontaneous process). The calculated value of ΔH° (+ ve 79.9 kJ/mol) is implicit in the endothermic characteristics of the process.

**Table 11:** Thermodynamic Parameters of the removal of MB Dye by using NHGP.

Temperature (K)	K_d_	ΔG° (kJ/mol)	ΔH° (kJ/mol)	ΔS° (J/mol K)
298	0.15	4651.18892	79.726	252.94
315.5	1.33	− 748.0434725		
333	4.44	− 4126.969062		

Moreover, the adsorption process may be classified as a chemical process or, a physical process, or may be operated by the two mechanisms simultaneously^77^. The Δ*H* value for van Edward force, hydrogen bond, ligand exchange, dipole interaction, and chemical bond is 4–10, 2– 40, ≈ 40, 2–29, and > 60 kJ⋅mol^-1^, respectively^78^. The Δ*H* value of the adsorption of MB onto synthesized NHGP was 79.7 kJ.mol^-1^, indicating the adsorption by a chemical bond.

The adsorption capacity of NHGP synthesized in the current study for methylene blue is compared with the adsorption capacity of different composite geopolymers as shown in Table 12. According to the data below, it can be indicated that the NHGP produced from fired brick waste (Homra) is a suitable sorbent for MB removal from economic and environmental perspectives.

**Table 12:** Comparison between Different Geopolymer Adsorbents for MB Adsorption Uptake.

Dye	Type of geopolymer composite	Adsorption capacity, mg/g	Refs.
Methylene Blue	CTAB-Cu_2_O/TiO_2_ Geopolymer composite	20.11	^79^
	Fly ash geopolymer (FAG)	37.04	^80^
	Fly ash geopolymer	15.4	^81^
	Volcanic ash-metakaolin based geopolymer (GP0 and GP10 and GP30)	13.9, 14 and 14.1	^82^
	Metakaolin-based geopolymer (MKG)	43.48	^24^
	New graphitic carbon nitride/geopolymer	170.9	^41^
	Fly ash–based geopolymer spheres	30.1	^34^
	Magnetite /geopolymer composite MGP	76.34	^83^
	Pyrophyllite clay porous geopolymer	64.1	^84^
	Coal fly ash geopolymer	50.7	^85^
	Present study	80.65	

### Desorption and reusability for MB adsorption

**Figure 17:**
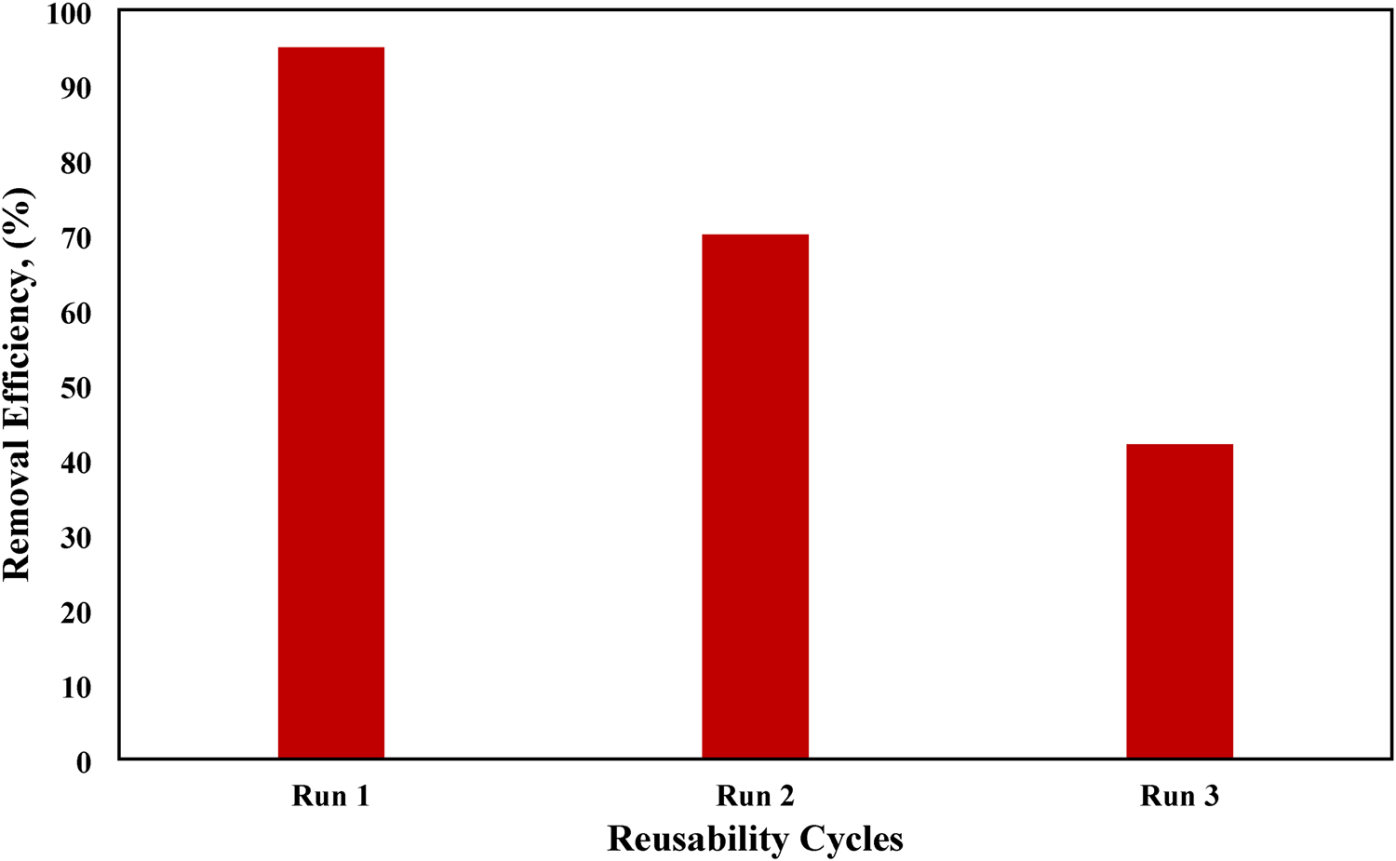
Reusability of NHGP.

The commonly employed alcohol-washing method was first used for the regeneration of NHGP. During alcohol washing, the color of the solution turned from transparent to blue, indicating the desorption of MB from the spent adsorbent. After three times washing, the regenerated NHGP was used for the next adsorption run. As shown in Fig. 17, qe of MB decreased sharply in the next adsorption runs. The decreased amount of adsorption was attributed to residual MB on the surface of NHGP. As discussed in the “Effect of pH” section, although electrostatic repulsion existed between MB and the adsorbent surface at lower pH values, hydrophobic adsorption, and electron donor–acceptor (EDA) interaction could still lead to the adsorption of MB. In other words, the adsorbed MB could not completely desorb in the regeneration process, and also 20% of the adsorbent weight was lost during the regeneration. In addition, although repeated washing could enhance the desorption of MB, more spent washing alcohol and water would be produced, which needs further treatment. Thus, the regeneration method was not favorable from a practical viewpoint that decreased the removal efficiency from 95% at the first use to 70% at the second use and finally to 42%.

## Conclusion

This work effectively synthesized a nano-geopolymer from waste-fired brick (Homra) as a novel solid particle adsorbent for wastewater purification, offering an unanticipated opportunity to control the geopolymer materials' structure for high-efficiency dye adsorption. The nano geopolymer was prepared using a 12M NaOH solution as an activator, with a 3:1 Na_2_SiO_3_/NaOH ratio. The nano-Homra geopolymer (NHGP) ideal SBET-specific surface area was 18.3658 m^2^/g, which offered enough channels for the mass transfer and adsorption processes. At the optimal conditions of 59 ℃ temperature, 254 mg/L initial concentration, and 163 min experiment duration, the novel nano-geopolymer adsorbent particles demonstrated high adsorption efficiency for MB dye, with a maximum adsorption capacity reaching 80.65 mg/g with a removal efficiency of 95.11%. Additionally, the test findings also demonstrated that the main external constraints affecting the entire adsorption process were the adsorbent concentration and experiment time. Additionally, the adsorbent's regeneration removal effectiveness was tested without causing a significant mass loss. The modified matrix structure of the nano-geopolymer was associated with the enhanced porosity and adsorption characteristics of the adsorbent. Because of their straightforward construction and practical treatment method, the resulting adsorbents were the predominant substitutes for more expensive or powder adsorbents. The novel nano-geopolymers have the potential to be used as adsorbent or carrier materials in various industrial applications.

## Data Availability

All data generated or analyzed during this study are included in this published article.
